# Rectal use of levetiracetam: best practice report for a stepwise approach for sustainable off-label decision making and treatment

**DOI:** 10.1007/s11096-023-01539-3

**Published:** 2023-02-08

**Authors:** Constanze Rémi, Alina Hermann, Elisabeth Krull, Franziska Ockert-Schön, Jan Rémi

**Affiliations:** 1 Department of Palliative Medicine, Munich University Hospital, LMU Munich, Marchioninistr. 15, 81377 Munich, Germany; 2 Hospital Pharmacy, Munich University Hospital, LMU Munich, Munich, Germany; 3 Zentrum für Ambulante Hospiz- und PalliativVersorgung München Land, Stadtrand und Landkreis Ebersberg, Oberhaching, Germany; 4 Hospital Pharmacy, A. ö. Krankenhaus St. Josef Braunau GmbH, Braunau, Austria; 5 Department of Neurology, Munich University Hospital, LMU Munich, Munich, Germany

**Keywords:** Clinical decision-making, Levetiracetam, Off-label use, Palliative care, Rectal drug administration

## Abstract

Off-label drug use is common practice in palliative care. It may pose a risk to the patient and benefit should outweigh harm. A decision and documentation aid for off-label use was developed to support practitioners in clinical practice off-label use. Using the example of the rectal administration of levetiracetam in three patient cases, the utilisation and benefits of the decision and documentation aid are presented and discussed. The rectal administration of levetiracetam clearly is an experimental treatment approach with little underlying evidence. To support and document the decision-making process for or against such an off-label use in clinical practice, it is helpful to have a structured approach in order to make this data comprehensible for a later point in time. Off-label use may be a permissible treatment alternative without underlying evidence, provided it takes place in a well-planned and well-monitored therapeutic setting and the benefits outweigh the potential risks.

## Background

Off-label use is common and necessary in medicine in general and palliative care in particular [1]. It describes a lack of official marketing authorisation for a specific indication or administration [2]. New or extended approval of a drug depends typically on a manufacturer’s initiative, where many factors besides the potential drug benefit are at play. The availability of approved drugs for an indication is therefore not necessarily determined by the available evidence, but certainly also to a large extent by economic interests. Off-label use involves risks for the prescriber at various levels: reimbursement, liabilities and drug safety among others [3]. Off-label use may pose a risk to the patient because it may not or insufficiently have been tested for the side effects of that particular situation.

Therefore, off-label use is not used lightly, but typically from a clinical knowledge that its potential benefits outweigh its potential harm. Before off-label use, approved therapy options should have been exhausted, or not be possible due to side effects, contraindications or maybe even economic considerations. The evidence should suggest a reasonable likelihood of a favourable therapeutic outcome. It should also be taken into account whether the off-label-use under consideration can rely on a profound evidence base or is an informed but new treatment. Ideally, the off-label use decision-making process is structured—particularly to determine the best possible treatment for each patient. On the one hand, this ensures that all relevant aspects are taken into account in the treatment planning. At the same time, the documentation of this decision-making process, as well as the subsequent therapy, can also help to comprehend the experiences made at a later point in time.

Using the antiepileptic drug levetiracetam as an example, we demonstrate a best practice report on how off-label use can be addressed in such a structured way in everyday palliative care. In palliative care, levetiracetam is commonly used for focal epilepsy [4], often secondary to a brain tumor. Its advantages in palliative care are relatively good tolerability, rapid achievement of maintenance dose and the availability of parenteral preparations. These non-oral routes of administration can be beneficial at end of life situations, when a patient is no longer able to swallow. Levetiracetam is approved for intravenous use and successful off-label subcutaneous use has been described previously [5–7], which is well stablished practice in general [8, 9]. However, a parenteral application cannot always be realised, e.g. in homecare. Then, alternative therapies must be discussed, either with alternative dosage forms, administration routes or other substances.

**Fig. 1:**
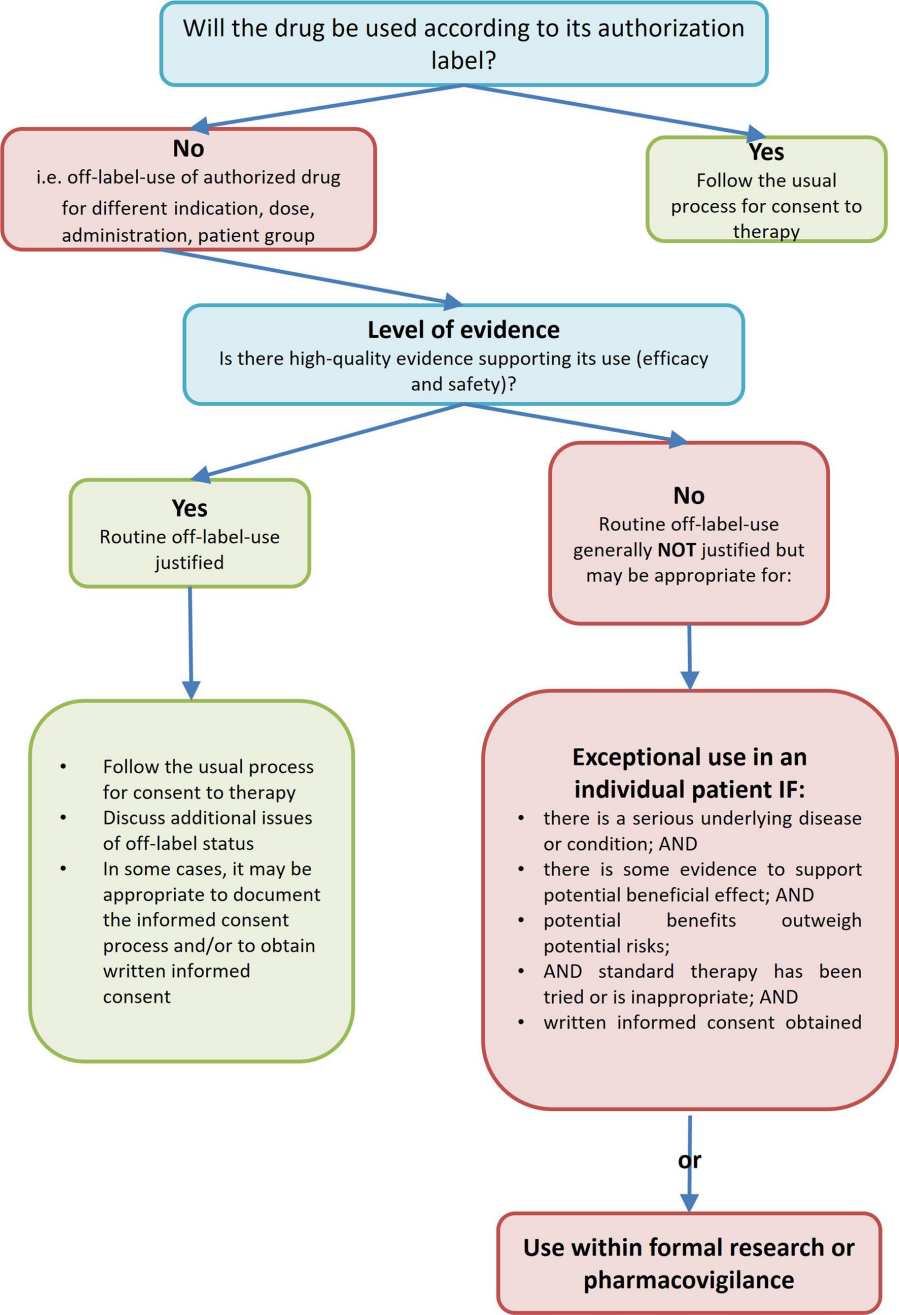
Decision-aid for off-label drug use

A modified version of the decision aid for off-label use proposed by Gazarian et al. [10] was developed by a pharmacist and a physician, evaluated by a panel of 14 experts from the fields of medicine, pharmacy and nursing (Fig. 1) and published in a general information brochure on off-label use in palliative medicine [11]. Here, we demonstrate the use of the decision and an accompanying documentation aid by using the rectal administration of levetiracetam as an example.

Based on the approach to off-label use in the general information brochure [11], a documentation aid was developed which takes up aspects of the decision aid once again. The decision and documentation aid, respectively, are intended to support practitioners in the decision-making process and documentation of off-label use and help to make it reproducible at a later point in time. Using the example of three patient cases for the rectal administration of levetiracetam outside the authorization, we demonstrate the decision and documentation aid (Table 1).

**Table 1:** Off-label use documentation aid with the case-specific information

	Patient 1	Patient 2	Patient 3
*Drug related information*			
Active ingredient and available product used off-label	Levetiracetam; suppositories compounded individually from original trademark	Levetiracetam; suppositories compounded individually from original trademark	Levetiracetam; suppositories compounded individually from original trademark
(Off-label-) indication	Epilepsy (tonic–clonic seizures) caused by brain tumor	Epilepsy caused by brain tumor	Epilepsy caused by brain tumor
Previous treatment attempts (active substance or reason why they are no longer used)	Levetiracetam p.o. 2000 mg twice daily Lacosamide 50 mg p.o. twice daily Pantoprazole p.o. 40 mg once daily Dexamethasone p.o. 8 mg once daily Oral medication stopped due to dysphagia. Parenteral administration difficult in the homecare sector	Levetiracetam p.o. 1000 mg twice daily Oral medication stopped due to dysphagia. Parenteral administration difficult in the home care sector	Levetiracetam p.o. (oral suspension) 1000 mg twice daily Oral medication stopped due to dysphagia. Parenteral administration difficult in the home care sector
Type of off-label use (e.g. route of administration, dose, indication, etc.)	rectal Use of levetiracetam	Rectal use of levetiracetam	Rectal use of levetiracetam
Basis for decision-making	Interdisciplinary case discussion based on Slikkerveer et al. [12], Gallentine et al. [13], Dunteman et al. [14]	Interdisciplinary case discussion based on Slikkerveer et al. [12], Gallentine et al. [13], Dunteman et al. [14]	Interdisciplinary case discussion based on Slikkerveer et al. [12], Gallentine et al. [13], Dunteman et al. [14]
*Patient-related data*			
Age and primary diagnosis	29 years Astrocytoma	57 years Glioblastoma	61 years Glioblastoma
Current medication (as complete as possible)	Levetiracetam rectally 2000 mg twice daily Morphine s.c. (dose unknown)	Levetiracetam rectally 750 mg three times daily	Levetiracetam rectally 1000 mg twice daily
*Monitoring of treatment*			
Which therapeutic goal is to be achieved with the treatment?	Seizure control	Seizure control	Seizure control
Monitoring parameters for side effects (including monitoring interval and responsibilities)	rectal irritation (upon administration; informal carer or nurse) treatment failure (daily; informal carer, nurse, physician)	rectal irritation (upon administration; informal carer or nurse) treatment failure (daily; informal carer, nurse, physician)	rectal irritation (upon administration; informal carer or nurse) treatment failure (daily; informal carer, nurse, physician)
Monitoring parameters for effect (including monitoring interval and responsibilities)	seizure frequency (daily; informal carer, nurse, physician)	seizure frequency (daily; informal carer, nurse, physician)	seizure frequency (daily; informal carer, nurse, physician)
*Informed consent*			
Information for patient or authorized representative	Parents (official representatives) of the patient gave informed consent	wife (official representative) of the patient gave informed consent	Patient gave informed consent
*Treatment effect*			
Was the treatment goal achieved (effect strength 0 = no effect until 10 = treatment goal completely achieved)?	10; no more seizures observed	10, no more seizures observed	10, no more seizures observed
If applicable, details of serumlevels incl. time of collection	No serum levels (lack of venous access)	No serum levels (lack of venous access)	Serum trough levels levetiracetam p.o. (2 × 1000 mg): 109 umol/l
			Serum trough levels levetiracetam rectal (2 × 1000 mg): 122 umol/l
In which time frame was the effect observed?	No seizures over a period of 2 weeks (until death of the patient)	No seizures over a period of 7 days (until death of the patient)	No seizures over a period of 9 days (until the patient was discharged from the hospital)
What side effects (positive and negative) occurred?	None	None	None
Will the treatment be continued?	Not applicable, patient died	Not applicable, patient died	Not applicable; patient died
If no: why not?			

## Case presentation

### Case 1

This 29-year-old woman had a relapse of a frontotemporal astrocytoma 2 months before admission, last treated with bevacizumab. She had developed progressive cognitive deficits and was eventually admitted to an inpatient hospice. Levetiracetam 2 × 1000 mg and dexamethasone 8 mg did not provide good seizure control (6 tonic–clonic seizures per day). However, regular and reliable medication intake was questionable. Nevertheless, lacosamide at 2 × 200 mg was initiated. Although it was poorly tolerated (cognitive impairment), she was seizure-free. The medication was continued until the patient developed dysphagia and oral medication intake was not feasible anymore. She had no venous access and her legal guardians refused to have a new one placed. Since the patient also complained of headaches, morphine s.c. was initiated. The attending physician decided against the parenteral administration use of dexamethasone, lacosamide and levetiracetam as subcutaneous infusions. After an interdisciplinary discussion of the case guided by the decision aid and including a patient-individual benefit-risk assessment, rectal therapy with levetiracetam was initiated (off-label use). Based on the available data [12], an equivalent dose (1:1) was used. Rectal suppositories had to be compounded from commercially available levetiracetam tablets (levetiracetam in mixed fatty acid glycerides (Witepsol® or Suppocire®) according to the Münzel-procedure [15], because a pure levetiracetam compound is not available in Germany. A determination of the levetiracetam serum concentrations was not possible due to the poor venous status of the patient. The suppositories were well tolerated. No further epileptic seizures occurred. Regular use of the suppositories by nursing staff was ensured, which may also explain the effectiveness of monotherapy. The rectal administration of levetiracetam was continued at unchanged doses for 2 weeks until the death of the patient.

### Case 2

This 57 year old male had epilepsy secondary to glioblastoma. Seizures were initially well controlled with levetiracetam 1000 mg p.o. twice daily. With the progression of the disease, the patient developed dysphagia and was unable to take his oral medication. Homecare was provided by a specialized palliative homecare team. Parenteral drug therapy could not be reliably realized in this patient-individual setting. Accordingly, an alternative route of application or an alternative drug had to be found. Further therapy planning was based on an interdisciplinary assessment of the case, using the decision aid. Rectal midazolam or diazepam were rejected due to sedative effects. Since the therapy with oral levetiracetam was well tolerated and effective, a rectal therapy trial with levetiracetam seemed justified after patient-specific risk–benefit analysis. The oral dose of levetiracetam was changed to 3 × 750 mg rectally, instead of the suggested dosing ratio of 1:1 (based on [12]). The dosing regimen was changed, because the compounding pharmacy could only provide preparation for levetiracetam 750 mg suppositories. The compounding was done by the same procedure as described above. The treatment was well tolerated, no epileptic seizures were observed during the treatment period. Blood level could not be analysed as there was no venous access. The rectal administration of levetiracetam was carried out for 7 days until the patient died.

### Case 3

This 61 year old man had been diagnosed with glioblastoma of the left frontal lobe. The secondary epilepsy was treated with levetiracetam. With the progression of the disease, swallowing became increasingly challenging. When oral administration was no longer reliably possible, the palliative homecare team decided to assess other therapy options with the help of the decision aid. The parenteral administration of levetiracetam was hardly feasible in the patient's care environment. Benzodiazepines were rejected because of their sedative effects. The rectal administration of levetiracetam was acceptable to the patient and his caregivers. The oral dose of 1000 mg twice daily was changed to a 1:1 rectal dose. Rectal 500 mg suppositories were compounded as already described in case 1. Levetiracetam serum concentrations before the switch were 109 µmol/l. On day 3 after rotation, serum concentrations of levetiracetam were at 122 µmol/ l (reference: 30–176 µmol/l [16]). The treatment was successfully continued without further seizures until the patient died 2 weeks later. There were no signs of side effects.

## Summary of cases

These three patients were cared for by independent palliative care teams and in different settings. The exceptional off-label use of rectal levetiracetam seemed justified despite the lack of high-quality evidence for this route of administration

- *Serious condition* all patients were suffering from a serious underlying condition.
- *Evidence* the available data suggest that levetiracetam is well absorbed from the rectum. It is not necessary to prove good efficacy of levetiracetam per se.
- *Risk–benefit–ratio* the potential benefit was the continuation of care in the home environment. The alternative treatment with benzodiazepines would have had a higher potential for non-desirable side effects (sedation) in the current context. The potential risks were addressed by various measures: emergency medication in the form of benzodiazepines was always available, and the patient was also monitored for local side effects; in addition, the observation of systemic side effects (not specific to rectal intake) was used as an indicator of overdosing. Changes in seizure frequency served as a surrogate parameter for possible underdosing. In the case of the third patient, blood levels were also determined.
- *Informed consent* in all three patient cases, either the patient or, if this was no longer possible, the legal guardians gave informed consent to the therapy. This also included information about the experimental character of this off-label use, potential therapy alternatives and expected effects or side effects. Consent to the therapy was obtained before the start of the therapy.

## Discussion

Off-label use is common in palliative care [1], when it is well established and known to be efficacious (e.g. opioids in dyspnea). Without underlying evidence, it should only be applied in a well-planned and well-monitored setting and when potential benefits outweigh potential risks. Rectal administration of levetiracetam represents such a potential alternative, without approval and with little evidence of its use. In healthy volunteers, receiving a single dose of 500 mg rectally, approximately the same bioavailability as 500 mg p.o. had been shown. However, absorption from the rectum was slower and peak levels were lower than after oral administration [12]. The effect of levetiracetam was investigated in eleven children with refractory status epilepticus; one child also received levetiracetam rectally [13]. Minor rectal bleeding was observed in this child, which the authors associated with frequent rectal administration rather than the actual medication. A response of the status epilepticus to this medication was not observed.

Rectal administration of levetiracetam was effective in the treatment of neuropathic pain in three palliative care patients [14]. Patients received levetiracetam 1000 mg every 12 h in a water-soluble lubricant. Blood levels were measured in one patient. Exact concentrations are not described. Based on the measurements, the authors assumed adequate resorption of levetiracetam from the rectum. In the other two patients, blood levels were not determined.

These available data can support a pragmatic individualized decision for or against levetiracetam rectally. However, this treatment approach is highly experimental, therefore carrying a greater potential risk of harmful effects since no controlled studies are available. Irrespective of available evidence, clinical scenarios are common in palliative care, where only off-label use is possible. Treatment should then only be considered after a thorough risk–benefit analysis, including possible alternatives. This risk–benefit analysis may have to be repeated, e.g. when the patients’ performance status changes. In our cases the rectal route was used over a relatively short period of time; a longer treatment period might already lead to a different risk–benefit analysis.

A structured approach supports and documents the decision-making process, which will make the data comprehensible and comparable at a later point in time. With the example of our three cases, a structured decision-making process and uniform therapy documentation for the rectal use of levetiracetam was demonstrated. On one hand, this ensures that all relevant aspects are taken into account in the planning and implementation of therapy. On the other hand, off-label use experiences can be used in a sustainable manner.

It seems that awareness of the potential consequences of off-label use in palliative care is still low, including little concern about the lack of evidence regarding treatment benefit [17]. A structured decision-making process can help validate off-label treatment from a therapeutic perspective. One challenge is that there is not unequivocal cut off for the decision for or against off-label use, making it user-subjective to a certain degree. The publication of three cases is not an evidence base as mentioned above, but a first structured data collection. The demand for evidence-based therapy in end of life care is not self-serving, but is intended to help make drug therapy as safe and effective as possible. Making standardized, reproducible decisions and monitoring therapy can already contribute to a more conscious and sustainable use of off-label drugs in individual institutions.

The collection of off-label hospital pharmacy use experiences at one central site can be an important step towards the creation of an evidence base. Such an office should not only gather experience, but also support professionals in decision-making and provide therapy guidance, e.g. in the form of use-specific documentation aids. From this first important starting point, further necessary research activities can then be planned. At the same time, the central collection can also help to pool negative experiences in order to advise against certain therapy approaches at an early point in time or to be able to name more suitable therapy alternatives. The Central Office Off-Label-Use in Palliative Care at the Department of Palliative Medicine, LMU Klinikum, should fulfil this task, at least for the time being in German-speaking countries.

## Conclusion

Off-label use is a relevant topic in palliative medicine and has already entered the consciousness of many prescribers. What is now in demand is support in everyday clinical practice with a direct bridge to the creation of evidence. The presented decision and documentation aid helps to ensure that all relevant aspects are taken into account in the planning and implementation of therapy. On the other hand, off-label use experiences can be used in a sustainable manner. Our best practice model can help to build this bridge by providing a systematic approach for clinicians in evaluating the appropriateness of off-label drug use in palliative care . It can also help to start filling knowledge gaps, to inform future treatment decisions and to set the future research agenda.
